# Determining the relationship of food neophobia, digital addiction, body image perception on social media with dietary inflammatory index among adolescents

**DOI:** 10.3389/fpubh.2025.1683764

**Published:** 2025-10-30

**Authors:** Rumeysa Albayrak, Yagmur Demirel Ozbek

**Affiliations:** ^1^Department of Nutrition and Dietetics, Faculty of Health Sciences, Uskudar University, Istanbul, Türkiye; ^2^Department of Nutrition and Dietetics, Faculty of Health Sciences, Recep Tayyip Erdogan University, Rize, Türkiye

**Keywords:** nutrition, adolescents, food neophobia, digital addiction, dietary inflammatory index, social media

## Abstract

**Background:**

Adolescence is a critical period in which both physical and psychological development accelerates and foundational dietary behaviors are established. This study aims to examine the relationships of food neophobia, digital addiction, body image perception on social media with the dietary inflammatory index (DII) among high school adolescents.

**Method:**

This cross-sectional and descriptive study was conducted with a total of 354 high school students, including 200 females and 154 males. Data were collected through questionnaires and face-to-face interviews. The questionnaire included sections on general information, dietary habits, the Food Neophobia Scale (FNS), the Child and Adolescent Digital Addiction Scale (CADAS), the Appearance-Related Social Media Consciousness Scale (ASMC), and a 3-day dietary intake record. Data analysis was performed using IBM SPSS version 25.

**Results:**

The mean DII score was 3.23 ± 1.66, the mean FNS score was 38.33 ± 10.81, the mean CADAS score was 35.82 ± 11.53, and the mean ASMC score was 41.31 ± 17.71. Participants who skipped meals had significantly higher DII, CADAS, and ASMC scores (*p* < 0.05). Regular breakfast consumption was associated with lower DII and CADAS scores (*p* < 0.05). A positive correlation was found between DII and CADAS, as well as between CADAS and ASMC. FNS scores showed a negative correlation with CADAS only among female participants.

**Conclusion:**

To support healthy adolescent development and mitigate the risks posed by the digital age, it is recommended to design and implement comprehensive intervention programs based on school, family, and individual levels, utilizing multidisciplinary approaches.

## 1 Introduction

Adolescence is a critical stage of life situated between childhood and adulthood, during which individuals experience rapid biological, psychological, and social development (1). In this period, adolescents’ energy and nutrient requirements increase, and their eating behaviors are shaped by a variety of multidimensional factors, including personal attitudes, environmental conditions, and social influences (2).

Adolescents’ dietary behaviors are influenced by physiological needs, food availability, and cultural norms, as well as by food neophobia, defined as the reluctance to try unfamiliar foods. This behavior leads to avoidance of unfamiliar food items and, consequently, a reduction in dietary variety (3). Food neophobia has been associated with inadequate consumption of essential and healthy food groups such as vegetables, fruits, fish, and dairy products, which may result in deficiencies in key micronutrients such as vitamins, minerals, and dietary fiber (4–6). These deficiencies, especially during the growth period, may contribute to long-term health problems (7).

In addition to individual characteristics and psychological tendencies, environmental factors play a significant role in shaping adolescents’ dietary habits; in particular, the increased use of digital platforms and screen time represents a major component of these environmental influences (8, 9). The increasing integration of digital devices into daily life due to advancing technology has also negatively affected adolescents’ nutritional habits. The growing time spent on social media, digital games, and online content can lead to delayed mealtimes, reduced physical activity, and a rise in unhealthy food choices (10, 11). Recent studies have demonstrated that digital addiction in adolescents is significantly associated with meal skipping, poor dietary quality, and unhealthy eating patterns (12, 13). These adverse dietary behaviors, often accompanying digital dependency, may elevate the inflammatory potential of the diet, reflected in increased levels of circulating inflammatory biomarkers and a decrease in overall diet quality (14).

The widespread use of social media has also been shown to influence body image perception among adolescents. The abundance of appearance-focused and image-centric content on these platforms can intensify concerns about physical appearance and contribute to lower levels of body satisfaction (15). This tendency for social comparison on social media is particularly prevalent among female adolescents, leading to increased body dissatisfaction, irregular eating habits, meal skipping, and engagement in unhealthy dieting practices (16, 17).

Psychosocial factors such as food neophobia, digital addiction, and appearance-related concerns on social media not only impact physical health but may also play a role in determining the inflammatory potential of dietary intake, as measured by the Dietary Inflammatory Index (DII) (18–21).

This study was designed and conducted to examine the relationships between food neophobia, digital addiction, body image perception on social media, and the Dietary Inflammatory Index among adolescents.

## 2 Materials and methods

### 2.1 Study population and research design

This cross-sectional study was conducted at Kadıköy Anatolian Imam Hatip High School for Boys and Çamlıca Anatolian Imam Hatip High School for Girls in Istanbul from January to March 2025. Participants were students aged 14–18 who consented to take part in the research. Sample size was calculated assuming an effect size of f^2^ = 0.20 (small/medium), a Type I error of *α* = 0.05, and statistical power of 1 – *β* = 0.80, yielding a minimum required sample size of 299. Ethical approval was obtained on December 31, 2024 (approval number: 61351342/020–714). A total of 354 students who met inclusion criteria voluntarily participated. Written informed consent was obtained from all participants’ parents or legal guardians. The study was conducted in accordance with the principles of the Declaration of Helsinki.

Data were collected through face-to-face interviews using a structured questionnaire. The questionnaire included sections on sociodemographic characteristics, general information, dietary habits, the Food Neophobia Scale (FNS), the Child-Adolescent Digital Addiction Scale (CADAS), the Appearance-based Social Media Consciousness Scale (ASMC), and a three-day food consumption record. The dietary inflammatory index (DII) was computed based on the three-day food consumption records.

### 2.2 Data collection tools

#### 2.2.1 Anthropometric measurements

##### 2.2.1.1 Body weight

Body weight (kg) was measured using a portable digital scale accurate to 0.1 kg, with participants wearing light clothing and no shoes. Measurements were recorded in the questionnaire form (22).

##### 2.2.1.2 Height

Height was measured using a non-stretchable measuring tape while participants stood with their back against the wall, feet together, heels, hips, and head touching the wall, and the head positioned in the Frankfurt horizontal plane. Measurements were recorded in the questionnaire form (23).

##### 2.2.1.3 Body mass index (BMI)

Age- and sex-specific BMI-Z scores were determined based on the World Health Organization’s reference standards for children and adolescents aged 5 to 19 years, using the WHO AnthroPlus software program (24).

#### 2.2.2 Food Neophobia Scale (FNS)

The Food Neophobia Scale (FNS) was developed by Pliner and Hobden in 1992 to measure individuals’ fear or reluctance toward trying new or unfamiliar foods (25). The Turkish adaptation of the scale was carried out by Duman et al. in 2020 (26). The scale consists of a total of 10 items—5 positively worded and 5 negatively worded—related to food and eating behaviors. The FNS is evaluated using a 7-point Likert scale (7 = Strongly agree, 6 = Agree, 5 = Slightly agree, 4 = Neutral, 3 = Slightly disagree, 2 = Disagree, 1 = Strongly disagree). Items 1, 4, 6, 9, and 10 are reverse-scored (R). The interpretation of the scores is based on the mean (X̄) and standard deviation (±SD). Participants scoring below <X̄ − 1SS are considered neophilic (favorable toward new foods), those scoring above >X̄ + 1SS are considered neophobic (averse to new foods), and those with scores within the X̄ ± 1SS range are considered neutral in their attitude toward trying new foods.

#### 2.2.3 Child-Adolescent Digital Addiction Scale (CADAS)

The Child-Adolescent Digital Addiction Scale (CADAS) is an instrument developed by Seema et al. (27) to assess addictive behaviors related to digital device use among adolescents aged 11 to 19. The Turkish adaptation and the validity and reliability studies of the scale were conducted by Türk et al. in 2024 (10). The scale consists of 10 items that measure feelings and behaviors associated with digital device usage. Participants respond to these items using a 7-point Likert scale (1 = Never, 2 = Very Rarely, 3 = Rarely, 4 = Sometimes, 5 = Frequently, 6 = Usually, 7 = Always). The total possible score ranges from 10 to 70, with higher scores indicating a greater level of digital addiction.

#### 2.2.4 Appearance-Based Social Media Consciousness Scale (ASMC)

The Appearance-Based Social Media Consciousness Scale is designed to assess adolescents’ perceptions of their appearance on social media. The scale was originally developed by Choukas-Bradley et al. (28) and was adapted into Turkish by Yıldırım et al. in 2022 (29). It consists of 13 items evaluating individuals’ thoughts and behaviors related to their social media photos. Participants respond to each item using a 7-point Likert scale (1 = Never, 2 = Almost Never, 3 = Rarely, 4 = Sometimes, 5 = Often, 6 = Almost Always, 7 = Always). The total score ranges from 13 to 91, with higher scores indicating greater awareness of appearance on social media.

#### 2.2.5 Dietary inflammatory index (DII)

The Dietary Inflammatory Index (DII) is a literature-based scoring system developed to assess the potential inflammatory impact of an individual’s diet (30). The DII was initially developed by Cavicchia et al. in 2009, encompassing 42 dietary parameters to evaluate their inflammatory potential (31). In 2014, Shivappa et al. updated the index to include 45 dietary components reflecting the diet’s influence on inflammation (32).

The DII can be calculated using 24-h dietary recall interviews and food consumption records (33). In this study, the DII was computed based on participants’ three-day dietary records. These records were analyzed using the BeBiS Version 9 software, and the average intake values were used in the calculation. The scoring methodology developed by Shivappa et al. (32) was applied. A total of 34 dietary components were included in the calculation: energy, protein, carbohydrate, total fat, saturated fat, monounsaturated fat, polyunsaturated fat, n-3 fatty acids, n-6 fatty acids, cholesterol, fiber, caffeine, vitamins A, D, E, C, beta-carotene, thiamin, riboflavin, niacin, folic acid, vitamin B6, vitamin B12, iron, magnesium, selenium, zinc, garlic, ginger, turmeric, onion, green/black tea, pepper, and thyme. Due to limitations in the BeBiS software, some components (eugenol, trans fatty acids, flavan-3-ols, flavones, flavonols, flavanones, anthocyanidins, and isoflavones) could not be calculated; alcohol was also excluded since it was not consumed by the participants.

To compute the DII, z-scores were calculated for each nutrient based on participants’ average daily intake. These z-scores were then converted into centered percentile scores to normalize the distribution. Each percentile score was multiplied by its corresponding “inflammatory effect score,” as defined by Shivappa et al., to obtain a value for each component. The sum of all component scores constituted the individual’s overall DII score. Since there is no universally accepted cut-off point for interpreting DII scores, previous studies have categorized DII scores into quartiles for statistical analysis (21, 34–36). In this study, DII scores were sorted in ascending order and divided into four quartiles: Q1 (*n* = 89), Q2 (*n* = 89), Q3 (*n* = 88), and Q4 (*n* = 88). The first quartile (Q1) represents the most anti-inflammatory dietary pattern, and the dietary inflammatory burden increases progressively from Q1 to Q4. The DII score ranges for each quartile were as follows: Q1 ≤ 2.13, Q2 = 2.14–3.36, Q3 = 3.37–4.54, and Q4 ≥ 4.55.

### 2.3 Analysis of data

The data obtained from the questionnaires administered to the participants were first digitized using Microsoft Excel and then analyzed using IBM SPSS for Windows, version 25.0. The normality of the measurement tools was assessed based on skewness and kurtosis values. According to the results, some variables met the assumption of normal distribution, while others deviated from it. For variables with a normal distribution, comparisons between two independent groups were conducted using the Independent Samples t-test, while comparisons among more than two independent groups were performed using One-Way Analysis of Variance (ANOVA). When the ANOVA test revealed statistically significant differences among groups, Bonferroni-adjusted post-hoc tests were applied to determine the source of the differences. In cases where the data did not meet the normality assumption, the Kruskal–Wallis H test was used to compare more than two groups. When significant differences were identified, Bonferroni-corrected post-hoc analyses were conducted to determine which groups differed. To assess relationships between continuous variables, Pearson correlation analysis was used for normally distributed variables, and Spearman correlation analysis was applied for those that did not meet the normality assumption. Differences between categorical variables were examined using the Chi-square (χ^2^) test. A *p*-value of less than 0.05 was considered statistically significant, and all results were evaluated within a 95% confidence interval.

## 3 Results

General descriptive information about the participants is presented in Table 1. Among the 354 students who participated in the study, 56.5% were female and 43.5% were male. A total of 45.8% of the students reported consuming 2 main meals per day, while 46.9% reported having 3 main meals daily. It was found that 60.5% of participants skipped at least one main meal, with breakfast being the most frequently skipped meal (69.6%). As for eating out, 82.3% of participants preferred fast-food restaurants. The majority of participants (50.2%) stated that they preferred snacks such as chocolate and candy during between-meal times. According to the BMI-Z score and HFA-Z score (height-for-age Z-score) classifications, the majority of the adolescents in the study were within the normal range. According to the Food Neophobia Scale, the majority of participants (68.4%) were in the neutral group regarding new foods.

**Table 1:** Descriptive characteristics of the participants.

	*n*	%
Gender		
Female	200	56.5
Male	154	43.5
Grade level		
9th grade	157	44.4
10th grade	80	22.6
11th grade	88	24.9
12th grade	29	8.2
Weekday TV/computer usage time		
1–2 h	117	33.1
3–4 h	124	35
5–6 h	52	14.7
More than 6 h	61	17.2
Weekend TV/computer usage time		
1–2 h	65	18.4
3–4 h	101	28.5
5–6 h	95	26.8
More than 6 h	93	26.3
Number of main meals per day		
1 main meal	10	2.8
2 main meals	162	45.8
3 main meals	166	46.9
≥4 main meals	16	4.5
Skipping main meals		
Yes	214	60.5
No	140	39.5
Skipped meal		
Breakfast	149	69.6
Lunch	53	24.8
Dinner	12	5.6
Breakfast consumption status		
I eat breakfast	127	35.9
I sometimes eat breakfast	98	27.7
I do not eat breakfast	129	36.4
Snack food preferences		
Fresh/dried fruits, oily seeds	86	28.4
Yogurt	30	9.9
Baked goods (pastry, bagel, etc.)	35	11.6
Chocolate, candy, wafers, etc.	152	50.2
Out-of-home eating location		
Fast food restaurants	275	82.3
Restaurants serving home-style meals	16	4.8
Bakeries	17	5.1
Other*	26	7.8
Food neophobia scale groups		
Neophilic (<27.52)	60	16.9
Neutral (27.52 ≤ − ≤ 49.14)	242	68.4
Neophobic (>49.14)	52	14.7
DII quartiles		
Q1 (≤2.13)	89	25.1
Q2 (2.14–3.36)	89	25.1
Q3 (3.37–4.54)	88	24.9
Q4 (4.55≥)	88	24.9
BMI-for-age Z-score		
Obese	29	8.2
Overweight	66	18.6
Normal	243	68.6
Thin	16	4.5
Height-for-age Z-score		
Very short/short	2	0.6
Normal	343	96.9
Tall	9	2.5

^*^Other: school cafeterias, canteens, dining halls and similar facilities; n: number of participants; %: percentage.

Anthropometric measurements and scale scores by gender are presented in Table 2. Male students had significantly higher mean height (176.60 ± 7.05 cm), body weight (71.87 ± 15.39 kg), and BMI (22.99 ± 4.51 kg/m^2^) compared to female students (163.12 ± 5.36 cm; 56.45 ± 9.29 kg; 21.18 ± 3.09 kg/m^2^) (*p* < 0.001).

**Table 2:** Distribution of anthropometric measurements, FNS, CADAS, ASMC, and DII scores by gender.

	Male		Female		T test	*p*
	X̄ ± SS	Median (min-max)	X̄ ± SS	Median (min-max)		
Height (cm)	176.60 **±** 7.05	177.00 (148.00–194.00)	163.12 **±** 5.36	163.00 (150.00–180.00)	**−19.747**	0.000**
Body weight (kg)	71.87 **±** 15.39	68.00 (43.00–115.00)	56.45 **±** 9.29	55.00 (37.00–96.00)	**−10.986**	0.000**
BMI (kg/m^2^)	22.99 ± 4.51	2.76 (16.61–40.04)	21.18 ± 3.09	20.70 (14.36–34.01)	**−4.280**	0.000**
BMI-Z	0.96 ± 1.26	1.02 (−2.95 − +3.02)	0.41 ± 1.23	0.52 (−3.07 − +2.88)	**−0.235**	0.000**
FNS	36.90 **±** 11.48	37.00 (10.00–70.00)	39.43 **±** 10.15	40.00 (11.00–63.00)	**2.189**	0.029*
CADAS	33.06 **±** 9.98	31.00 (13.00–70.00)	37.96 **±** 12.20	38.50 (10.00–70.00)	**4.152**	0.000**
ASMC	35.92 **±** 15.58	34.00 (13.00–91.00)	45.47 **±** 18.17	43.00 (13.00–91.00)	**5.319**	0.000**
DII	2.81 **±** 1.77	2.80 (−2.00–6.18)	3.55 **±** 1.50	3.59 (−0.25–6.41)	**4.201**	0.000**

FNS – Food Neophobia Scale; CADAS – Child-Adolescent Digital Addiction Scale; ASMC – Appearance-Related Social Media Consciousness Scale; DII – Dietary Inflammatory Index.

*p < 0.05; **p < 0.001. Bold values indicate variables that were significant in the model (*p* < 0.05; *p* < 0.01).

Food neophobia scores were significantly higher among females (39.43 ± 10.15) than males (36.90 ± 11.48) (*p* < 0.05). Similarly, scores for digital addiction (37.96 ± 12.20) and appearance-based social media consciousness (45.47 ± 18.17) were significantly higher among females compared to males (*p* < 0.001). In contrast, DII scores were significantly lower among males (2.81 ± 1.77) than females (3.55 ± 1.50) (*p* < 0.001) (Table 2).

The distribution of correlations between anthropometric measurements and scores from the FNS, CADAS, ASMC, and DII scales is presented in Table 3. There were statistically significant negative correlations between FNS scores and height (*r* = −0.130; *p* = 0.014), body weight (*r* = −0.166; *p* = 0.002), and HFA-Z score (*r* = −0.114; *p* = 0.006). CADAS scores were also negatively correlated with height (*r* = −0.126; *p* = 0.018) and weight (*r* = −0.136; *p* = 0.010). Similarly, ASMC scores showed statistically significant negative correlations with height (*r* = −0.117; *p* = 0.028) and weight (*r* = −0.171; *p* = 0.001). In addition, a statistically significant negative correlation was found between DII scores and height only (*r* = −0.177; *p* = 0.001).

**Table 3:** Correlation between participants’ anthropometric measurements and Their FNS, CADAS, ASMC, and DII scores.

	FNS		CADAS		ASMC		DII	
Height	r	**−0.130**	r	**−0.126**	r	**−0.117**	r	**−0.177**
	p	**0.014** ^*^	p	**0.018** ^*^	p	0.028*	p	**0.001** ^**^
Body Weight	r	**−0.166**	r	**−0.136**	r	**−0.171**	r	−0.081
	p	**0.002** ^**^	p	**0.010** ^*^	p	**0.001** ^**^	p	0.128
BMI	r	0.062	r	−0.038	r	−0.058	r	−0.008
	p	0.247	p	0.474	p	0.273	p	0.878
BMI-Z score	r	0.105	r	0.034	r	0.097	r	0.002
	p	0.051	p	0.514	p	0.068	p	0.973
HFA-Z score	r	**−0.114**	r	−0.031	r	0.044	r	−0.039
	p	**0.006** ^*^	p	0.566	p	0.410	p	0.464

FNS – Food Neophobia Scale; CADAS – Child-Adolescent Digital Addiction Scale; ASMC – Appearance-Related Social Media Consciousness Scale; DII – Dietary Inflammatory Index; BMI-Z score – BMI for age Z score; HFA-Z score – Height for age Z score.

*p < 0.05; **p < 0.01. Bold values indicate variables that were significant in the model (*p* < 0.05; *p* < 0.01).

The relationship of FNS, CADAS, ASMC, and DII scores with certain variables is shown in Table 4. As the number of daily main meals increased, DII scores significantly decreased (*p* < 0.05). Participants who consumed 2 main meals per day had higher DII scores compared to those who consumed 3 or ≥4 meals. In addition, participants who consumed 2 meals per day had significantly higher ASMC scores than those who consumed 3 meals (*p* < 0.05).

**Table 4:** Distribution of the relationship between FNS, CADAS, ASMC, DII scores and selected variables.

Variables	FNS	CADAS	ASMC	DII
	X̄ ± SS	X̄ ± SS	X̄ ± SS	X̄ ± SS
Number of main meals				
1 main meal	40.90 ± 11.30	35.40 ± 12.55	47.10 ± 20.63	4.23 ± 1.03
2 main meals	38.74 ± 11.02	37.40 ± 12.31	43.52 ± 17.60	3.60 ± 1.48
3 main meals	38.14 ± 10.05	34.21 ± 10.39	38.19 ± 16.03	2.91 ± 1.68
≥4 main meals	34.50 ± 15.30	36.88 ± 12.69	47.81 ± 27.34	2.19 ± 2.24
test value	0.954^F^	2.163^F^	**3.268** ^**F**^	**8.843** ^**F**^
*p*	0.415	0.092	0.036*	0.000*
post-hoc			**2 > 3**	**2 > 3,4**
Skipping main meals				
Yes	38.85 ± 11.07	37.19 ± 11.61	43.56 ± 18.14	3.54 ± 1.64
No	37.53 ± 10.37	33.74 ± 11.13	37.88 ± 16.53	2.76 ± 1.58
test value	1.126 ^t^	**2.783** ^**t**^	**2.984** ^**t**^	**4.424** ^**t**^
*p*	0.261	0.006*	0.003*	0.000*
Breakfast consumption status				
I eat breakfast	37.59 ± 10.90	33.55 ± 11.11	39.24 ± 17.08	2.80 ± 1.68
I sometimes eat breakfast	38.23 ± 10.44	35.73 ± 10.93	40.88 ± 17.22	3.36 ± 1.58
I do not eat breakfast	39.12 ± 11.01	38.13 ± 12.01	43.69 ± 18.53	3.55 ± 1.62
Test value	0.684^F^	**5.173** ^**F**^	2.077^F^	**7.347** ^**F**^
*p*	0.524	0.006*	0.127	0.001*
post-hoc		**1 < 3**		**1 < 2,3**
Out-of-home eating location				
Fast food restaurants	37.73 ± 10.47	36.92 ± 11.89	42.67 ± 17.79	3.28 ± 1.62
Restaurants serving home-style meals	33.88 ± 14.97	30.82 ± 6.61	33.29 ± 13.03	3.92 ± 1.47
Bakeries	40.11 ± 8.42	29.61 ± 9.92	36.50 ± 18.73	2.11 ± 2.39
test value	1.232^F^	**9.195** ^**F**^	**3.132** ^**F**^	**3.688** ^**F**^
*p*	0.309	0.001*	0.045*	0.040*
post-hoc		**1 > 2,3**	**1 > 2**	**3 < 1,2**
Daily water consumption amount				
0–500 ml	42.36 ± 7.73	40.21 ± 13.25	44.09 ± 19.40	3.55 ± 1.50
500–1,000 mL	39.29 ± 10.94	37.48 ± 11.14	42.20 ± 18.76	3.29 ± 1.70
1,000–1,500 mL	38.38 ± 10.73	35.72 ± 10.45	42.13 ± 18.31	3.31 ± 1.62
1,500–2000 mL	35.58 ± 11.04	33.09 ± 12.24	40.71 ± 16.59	3.08 ± 1.67
>2000 ml	37.56 ± 11.30	34.05 ± 11.24	37.89 ± 15.21	3.01 ± 1.72
test value	**2.536** ^**F**^	**3.060** ^**F**^	0.921^F^	0.804^F^
*p*	0.040*	0.017*	0.452	0.523
post-hoc	**1 > 4**	**1 > 4**		
BMI-Z score				
Obese	34.41 ± 10.64	34.66 ± 10.96	34.31 ± 16.90	3.42 ± 1.70
Overweight	38.11 ± 9.85	35.39 ± 10.64	41.86 ± 17.87	3.18 ± 1.61
Normal	38.62 ± 11.12	35.86 ± 11.52	41.52 ± 17.32	3.19 ± 1.66
Thin	41.94 ± 8.81	39.12 ± 16.06	48.62 ± 21.81	3.63 ± 1.89
test value	1.950^**F**^	0.571^**F**^	2.480^**F**^	0.510^**F**^
*p*	0.122	0.638	0.061	0.678

FNS – Food Neophobia Scale; CADAS – Child-Adolescent Digital Addiction Scale; ASMC – Appearance-Related Social Media Consciousness Scale; DII – Dietary Inflammatory Index.

^*^p < 0.05; F = One-way Analysis of Variance (ANOVA); t = Independent Samples t-test. Bold values indicate variables that were significant in the model (*p* < 0.05; *p* < 0.01).

Participants who skipped meals had significantly higher CADAS, ASMC, and DII scores compared to those who did not skip meals (*p* < 0.05). DII and CADAS scores were found to be lower in participants who regularly consumed breakfast compared to those who sometimes or never had breakfast (*p* < 0.05) (Table 4).

Participants who preferred fast-food restaurants had significantly higher CADAS and ASMC scores than those in other categories (*p* < 0.05). Moreover, participants who preferred bakeries/pastry shops had significantly lower DII scores than those who preferred fast-food restaurants (*p* < 0.05). Participants who consumed 0–500 mL of water per day had significantly higher FNS and CADAS scores compared to those who consumed 1,500–2000 mL of water per day (*p* < 0.05) (Table 4).

The distribution of the relationships between DII scores and FNS, CADAS, and ASMC by gender is shown in Table 5. Among all participants, there was a significant positive correlation between DII scores and CADAS scores (*r* = 0.138; *p* = 0.009), indicating that as the inflammatory potential of the diet increased, levels of digital addiction also slightly increased. A statistically significant positive correlation was also found between ASMC and CADAS scores (*r* = 0.394; *p* = 0.000), suggesting that an increase in appearance-related social media consciousness is associated with higher digital addiction.

**Table 5:** Distribution of the relationship of participants’ dietary inflammatory index scores, food neophobia scale, digital addiction scale, with social media appearance perception scale according to gender.

		DII	Food Neophobia Scale	Child-Adolescent Digital Addiction Scale	Appearance-Related Social Media Consciousness Scale
Total					
DII	r	1			
	p				
Food Neophobia Scale	r	0.008	1		
	p	0.874			
Child-Adolescent Digital Addiction Scale	r	**0.138**	−0.062	1	
	p	0.009*	0.241		
Appearance-Related Social Media Consciousness Scale	r	0.047	−0.092	**0.394**	1
	p	0.377	0.083	0.000**	
FEMALE					
DII	r	1			
	p				
Food Neophobia Scale	r	−0.084	1		
	p	0.238			
Child-Adolescent Digital Addiction Scale	r	0.126	**−0.203**	1	
	p	0.075	0.004*		
Appearance-Related Social Media Consciousness Scale	r	0.002	−0.129	**0.428**	1
	p	0.974	0.069	0.000**	
MALE					
DII	r	1			
	p				
Food Neophobia Scale	r	0.046	1		
	p	0.569			
Child-Adolescent Digital Addiction Scale	r	0.058	0.067	1	
	p	0.478	0.411		
Appearance-Related Social Media Consciousness Scale	r	−0.034	−0.131	**0.230**	1
	p	0.675	0.104	0.004*	

*p < 0.05; **p < 0.001. Bold values indicate variables that were significant in the model (*p* < 0.05; *p* < 0.01).

Gender-based subgroup analyses revealed a significant negative correlation between food neophobia and digital addiction among female participants (*r* = −0.203; *p* = 0.004). In the same group, there was a significant positive correlation between appearance-based social media consciousness and digital addiction (*r* = 0.428; *p* = 0.000), indicating that an increase in social media appearance concerns may raise digital dependency. Among male participants, there was also a statistically significant positive correlation between ASMC and CADAS scores (*r* = 0.230; *p* = 0.004) (Table 5).

## 4 Discussion

This study aimed to examine the relationships between food neophobia, digital addiction, appearance-based social media consciousness, and the Dietary Inflammatory Index (DII) among adolescents, and to reveal how these variables interact with dietary patterns. The findings indicate that psychosocial factors such as digital media use, food neophobia, and appearance-related perceptions on social media in adolescents have significant interrelationships and exert notable effects on dietary habits and body composition.

Globally, adolescents’ dietary habits are often characterized by the excessive consumption of high-energy, ultra-processed foods, breakfast skipping, fast food dependency, and insufficient intake of fruits and vegetables (37). To counterbalance these unhealthy dietary patterns and enhance dietary diversity, individuals need to be open to trying new foods (38). Food neophobia, defined as the tendency to avoid new or unfamiliar foods, may reduce dietary variety and overall diet quality, leading to inadequate intake of certain essential nutrients—particularly vitamins and minerals (7, 39, 40).

In our study, although food neophobia levels were found to be low in both sexes, female participants exhibited significantly higher levels compared to males. Consistent with our study findings, previous literature has reported that females exhibit higher levels of food neophobia compared to males (41, 42). During adolescence, dieting behavior, body image concerns, and body weight tend to be more prominent among girls than boys, which may lead to greater selectivity and reluctance toward new foods among female adolescents (43). These findings suggest that gender-related eating attitudes and social pressures that emerge during adolescence may contribute to a higher tendency toward food neophobia, particularly among females. In contrast to the results of our study, there are also studies in the literature that report no gender differences in food neophobia levels among adolescents (44–46). This inconsistency may be attributable to variations in the cultural, socioeconomic, and demographic characteristics of the sample groups used in different studies.

Findings based on the DONALD study suggest that elevated levels of food neophobia may be observed in both underweight and obese individuals (47, 48). In a study conducted among German adolescents, it was reported that individuals with low body weight had significantly higher food neophobia scores compared to their normal-weight peers (3). This finding supports the negative correlation identified in our study between body weight and FNS scores. The tendency of food neophobic individuals to follow restricted and low-energy diets may help explain the inverse relationship between food neophobia and body weight observed in our study (49, 50). Moreover, Xi et al. (51) found that children with higher levels of food neophobia exhibited significantly greater consumption of sugar-sweetened beverages. In this context, the higher food neophobia scores observed among participants with low water intake in our study may be interpreted as an indicative of poorer overall dietary quality and unhealthy lifestyle patterns.

The widespread use of digital media today has a significant impact on individuals’ dietary habits, particularly among adolescents, where it may contribute to both dietary restriction and unhealthy eating behaviors (52). The literature suggests that digital addiction reduces levels of physical activity (53), which in turn may lead to decreased energy expenditure and negative changes in body composition (54). The negative correlations identified in our study between digital addiction, and both body weight and height may be explained by the increase in sedentary behavior and irregular eating patterns associated with digital media use.

In our study, female participants were found to have significantly higher levels of digital addiction compared to their male counterparts. A review of the literature on gender-based differences reveals that males tend to be engaged more with game-oriented digital content, whereas females are more likely to use social media platforms (55–58). These differences indicate that digital addiction is not a unidimensional phenomenon, and that the types of addiction (e.g., gaming, social media, computer use, mobile phone use) are shaped by gender-specific usage patterns. Accordingly, the higher overall digital addiction scores observed among female adolescents in our study may be attributed, in part, to their greater engagement with social media. Therefore, it is suggested that digital addiction should be assessed through its subcomponents rather than as a holistic construct.

Our findings indicate that higher digital addiction is associated with skipping breakfast and main meals, inadequate water intake, and increased fast food consumption (Table 4). In line with these results, Kamran et al. (59) reported that university students with higher levels of internet addiction exhibited significantly higher rates of meal skipping. A large-scale study conducted in China suggested a potential association between digital addiction and breakfast skipping; however, it did not appear to have a significant effect on skipping lunch or dinner (60). Similarly, a study conducted among high school students in Egypt found that adolescents with high internet addiction were more likely to consume fast food and sugar-sweetened beverages, while showing less preference for home-cooked meals and healthier snacks such as fruits and vegetables (61). Behaviors such as staying up late and having irregular daily routines due to digital addiction may have disrupted participants’ eating schedules, leading to increased meal skipping. Moreover, adolescents who spend extended periods in front of digital devices may ignore thirst cues or replace water with sugary beverages, which could indirectly contribute to lower water intake and be associated with higher digital addiction scores in this group (62). Additionally, individuals who spend more time in digital environments may be more inclined to choose quick and convenient food options, thereby increasing their fast food consumption.

Digital addiction may lead individuals to spend more time on social media platforms, thereby increasing their exposure to appearance-based social comparisons and enhancing perceived pressure regarding physical appearance (63, 64). Studies have shown that increased time spent on social media, particularly among adolescent girls, is associated with higher levels of body surveillance, internalization of the thin ideal, and appearance-based comparisons, which in turn contribute to body dissatisfaction (65, 66). In our study, individuals with lower body weight exhibited higher appearance-based social media consciousness scores, which supports this pattern. Consistent with our findings, Jarman et al. (67) reported that adolescents with thin body types experienced greater body dissatisfaction as a result of appearance-based comparisons on social media. In the study conducted by Papapanou et al. (16), it was emphasized that the time spent on social media or the level of exposure to content affects perceived body satisfaction rather than objective measures such as an individual’s body mass index.

In our study, female participants were found to have significantly higher appearance-based social media consciousness scores compared to male participants. This finding is consistent with the existing literature. Mahon and Hevey (68) reported that girls tend to internalize social media content more strongly and exhibit more critical attitudes toward their own bodies. Similarly, Dahlgren et al. (69), in a study conducted among Norwegian adolescents, found a significant association between social media use, eating disorders, and appearance-related pressure, noting that female adolescents were more sensitive to these pressures. The frequent display of idealized images of thin female bodies on social media platforms may negatively influence adolescents’ body image and lead to increased levels of concern regarding social appearance (70). On the other hand, the lower perceptions of appearance among male participants on social media may be related to their engagement with less body-focused content. However, recent studies indicate that men can also be influenced by social media content due to body image expectations related to muscularity and physical strength (71–73). This suggests that appearance perceptions are not limited to women and that men can also be affected by the body norms presented on social media, albeit in different ways.

Research has shown that exposure to appearance-focused content on social media can increase body dissatisfaction, leading to negative eating behaviors such as social appearance anxiety and dietary restriction (74, 75). In support of these findings, our study revealed that participants who skipped meals had higher appearance-based social media consciousness scores. Furthermore, participants who preferred fast-food restaurants also scored higher on appearance-related social media consciousness. This may suggest that the “aesthetic and fast-consumption culture” frequently depicted in social media content may influence not only body image but also eating environment preferences. Hogue and Mills (76) proposed that exposure to aesthetically appealing food content on social media increases individuals’ awareness of their external appearance, which in turn may lead to changes in eating settings and behaviors.

Numerous studies in the literature have demonstrated that dietary intake can significantly influence systemic inflammation levels (77, 78). Systematic reviews indicate that anti-inflammatory dietary patterns—characterized by high intake of fiber, fruits and vegetables, whole grains, and omega-3 fatty acids—are associated with reductions in pro-inflammatory biomarkers such as CRP, IL-6, and TNF-*α*, whereas Western-style diets—rich in processed foods, refined carbohydrates, and saturated fats—have been shown to promote inflammation (14, 79). There is strong evidence linking high DII scores with both physiological health outcomes, such as obesity, metabolic syndrome, and inflammatory diseases, and psychosocial health outcomes, including impaired academic performance, depression, and anxiety (80–84).

In our study, female participants had higher DII scores compared to males. While this finding aligns with some studies in the literature (85, 86), other research has reported higher DII scores among males (87). The elevated DII scores observed among girls in our study may be attributed to several biological and behavioral factors, including a greater tendency to consume sweets and refined carbohydrate-rich foods, the influence of hormonal cycles on eating behavior, and social media-driven dietary patterns that may limit dietary variety. Nevertheless, inconsistencies in the literature may be explained by differences in dietary habits, hormonal influences, and methodological variability across studies.

In our study, participants who consumed fewer meals and particularly those who skipped breakfast were found to have higher DII scores. These findings suggest that irregular eating patterns may contribute to an increased inflammatory dietary load (88, 89). Consistent with our study findings, previous studies have reported that skipping breakfast is associated with higher DII scores (79, 90). This may be explained by the tendency of individuals who skip meals to make food choices throughout the day that are lower in nutritional value, lower in fiber, and higher in saturated fats, sugar, and refined carbohydrates, all of which contribute to elevated DII scores. These findings suggest that the daily food choices and meal patterns of young individuals may play a protective role in reducing the inflammatory potential of the diet.

In addition, a significant positive correlation was found between DII scores and digital addiction levels in our study (*r* = 0.138, *p* = 0.009). This finding suggests that as the pro-inflammatory potential of the diet increases, the tendency toward digital addiction also increases. Today, digital media use—especially among young individuals—has been shown to increase physical inactivity, which may lead to disruptions in dietary habits (75). Prolonged screen time may promote unstructured and poor-quality food intake, thereby fostering pro-inflammatory dietary patterns.

Additionally, a positive and significant correlation was found between digital addiction and appearance-based social media consciousness (*p* < 0.001). Fardouly and Vartanian (64) noted that individuals exposed to appearance-focused content on social media were more likely to engage in social comparison, which may, in turn, increase the amount of time spent online and raise the level of digital dependency. Similarly, Hogue and Mills (76), in a study conducted among young women, found that increased time spent on Instagram was significantly associated with greater body dissatisfaction and appearance-related comparison tendencies, particularly due to exposure to idealized body images.

Gender-based analyses in our study revealed a positive relationship between appearance-based social media consciousness and digital addiction among female participants (*p* < 0.001). Previous research suggests that adolescent girls’ perceptions of physical appearance may drive them to spend more time in digital environments, leading to increased tendencies for monitoring or comparing their appearance (28, 75). Among male participants, the correlation was also positive but weaker in magnitude (*p* < 0.05). The idealization of muscular and fit male bodies on social media platforms may contribute to similar forms of appearance-related pressure among male users as well (68).

Another noteworthy finding in our study was the negative correlation between food neophobia and digital addiction scores among female participants (*p* < 0.05). The literature reports that individuals with higher levels of food neophobia tend to exhibit behaviors such as avoiding external stimuli, reluctance toward novel experiences, and introversion (41, 91). On the other hand, studies on digital addiction indicate that individuals with higher levels of novelty seeking and extraversion tend to engage more intensely with the internet and social media (92–94). In this context, the negative relationship observed in our study suggests that neophobic tendencies may lead female adolescents to approach digital stimuli more cautiously, thereby contributing to lower levels of digital addiction.

Our findings carry significant implications not only from a scientific perspective but also for public health and educational practices. In this context, integrating nutrition education for adolescents with digital media literacy and psychosocial awareness programs, and reflecting these approaches in educational policies, is of particular importance. Furthermore, improving the nutritional quality of school cafeterias, conducting healthy eating workshops, and integrating nutrition education with digital technologies—such as mobile applications, online modules, or gamified programs—can be implemented to enhance student engagement and promote positive behavioral change.

Several aspects of this study enhance the reliability and contribution value of the findings. To our knowledge, this is among the first studies to holistically examine the relationships between food neophobia, digital addiction, appearance-based social media consciousness, and the DII in adolescents, thereby offering a novel contribution to the existing literature. The assessment based on three-day food consumption records provided a more accurate reflection of participants’ dietary patterns, thereby increasing the objectivity and validity of the nutritional data. Furthermore, the scales used in the study had previously undergone validity and reliability analyses, which supports the accuracy and trustworthiness of the collected data. The inclusion of four variables spanning different disciplines allowed for a comprehensive examination of the potential interactions between digital media use, body image perception, and eating behaviors. This multidimensional approach offers valuable insights for the fields of public health, nutrition, and psychology.

However, there are certain limitations that should be considered when interpreting the results and planning future research. Due to its cross-sectional design, causal inferences between the variables cannot be drawn. Additionally, the study focused solely on individual-level data and did not take into account environmental, socioeconomic, or parental influences that may also play a role. The data were collected from only two schools in Istanbul, which limits the representativeness and generalizability of the findings to the broader population.

## 5 Conclusion and recommendations

In our study, food neophobia, digital addiction, appearance-based social media consciousness, and DII scores were evaluated among high school adolescents, and the correlations between these variables were identified. These findings suggest significant associations between digital addiction, dietary behaviors, and psychosocial perceptions. Moreover, gender-based differences highlight the need for more sensitive and targeted approaches in intervention programs.

The increasing energy and nutrient requirements during adolescence make dietary habits a critical determinant of health. Unhealthy eating behaviors acquired during this period may increase the risk of inflammation-related obesity, metabolic syndrome, and chronic diseases in later life.

There is a need for multi-center intervention programs to support the healthy growth and development of adolescents, to positively influence their eating behaviors, and to help them cope with the risks brought by the digital age. As school settings are among the fundamental environments supporting both the social and cognitive development of young individuals, they offer a strategic foundation for the implementation of such interventions. In this context, school-based nutrition education should be strengthened with digital media literacy and psychosocial awareness modules.

It is further recommended to expand psychological support mechanisms aimed at helping adolescents develop a healthy body image, and to provide professional counseling services to improve their ability to cope with emotional eating, body dissatisfaction, and social media pressure. Within this scope, the integration of dietitians and guidance counselors in schools should be encouraged through a multidisciplinary approach.

This study is the first to comprehensively examine the variables of food neophobia, digital addiction, appearance-based social media consciousness, and the dietary inflammatory index among adolescents. Therefore, to better understand the causal relationships among these variables, further longitudinal and prospective studies with larger samples are needed.

## Data Availability

The raw data supporting the conclusions of this article will be made available by the authors, without undue reservation.
